# Prefrontal EEG Delta-Beta Coupling Marks Depressed State in Adult Siblings

**DOI:** 10.64898/2026.10.01.26364438

**Published:** 2026-10-02

**Authors:** Mina Elhamiasl, Lilian Y. Li, Felix A. Maldonado Osorio, Brent I. Rappaport, Stewart A. Shankman, Ivan Alekseichuk

## Abstract

Major depressive disorder (MDD) is marked by disorganization in electrophysiological brain networks, yet it remains unclear whether such disorganization reflects the state-dependent pathological effects or a trait-like vulnerability. We analyzed resting-state electroencephalographic (EEG) delta-beta phase-amplitude coupling (PAC) in two sibling cohorts. Current-MDD families included individuals with current MDD and their currently unaffected siblings (n=36), and remitted-MDD families comprised individuals with remitted MDD and their lifetime unaffected siblings (n=110). Delta-beta _high_ PAC was significantly elevated (p _corr_ ≤0.05) over the left-medial frontal region in participants with current MDD relative to their unaffected siblings. In the remitted-MDD families, no significant differences were observed. Regrouping all individuals irrespective of family ties into current MDD, remitted MDD, and never-depressed controls confirmed significantly elevated frontal delta-beta _high_ PAC at rest in the current-MDD subgroup compared to other subgroups. Overall, delta-beta _high_ coupling reflects the depressive state rather than a trait-like or familial vulnerability.

## Full Text Availability

The license terms selected by the author(s) for this preprint version do not permit archiving in PMC. The full text is available from the preprint server.

